# Subtype-specific enhancement of implicit statistical learning in migraine: insights from BOLD signal variability

**DOI:** 10.1186/s10194-026-02266-6

**Published:** 2026-02-19

**Authors:** Krisztián Kocsis, Laura Szücs-Bencze, Máté Csomós, Dániel Veréb, Lilla Horváth, Kisa Dominika, Krisztián Attila Szuly, Péter Faragó, Bernadett Tuka, Zsigmond Tamás Kincses, Nikoletta Szabó

**Affiliations:** 1 Department of Radiology, Albert Szent-Györgyi Health Centre, University of Szeged, Semmelweis u. 6, Szeged, 6725 Hungary; 2 Department of Neurology, Albert Szent-Györgyi Health Centre, University of Szeged, Szeged, Hungary

**Keywords:** Episodic migraine, Resting state functional MRI, BOLD signal variability, Temporal states, Implicit statistical learning

## Abstract

**Objectives:**

Migraine is associated with distinct cognitive alterations even during the interictal phase, yet the underlying mechanisms of implicit learning processes remain unexplored. Previous research has shown that the temporal variability of the blood-oxygen-level-dependent (BOLD) signal reliably predicts clinical symptoms in migraine. Building on these findings, the present study aims to investigate how implicit statistical learning processes relate to this resting-state functional imaging marker in episodic migraine. We hypothesized that implicit statistical learning would differ between migraine and non-migraine participants, and that these behavioral differences would be determined by group-specific patterns of resting state BOLD signal variability.

**Methods:**

This study employed a cross-sectional, case-control design. A total of 28 migraine patients (14 with aura, 14 without aura) and 22 healthy controls were enrolled in this study. Resting-state functional images were acquired during the interictal phase, and all participants accomplished the Alternating Serial Reaction Time task. Statistical learning performance was compared across the three groups using mixed-design ANOVAs. BOLD variability states were calculated based on their time-varying measures, and non-parametric permutation tests were used to examine group-level differences in functional network connectivity within these states. Subsequently, we analyzed group differences in the descriptive metrics of BOLD variability states. Finally, correlation analyses were performed to investigate how learning indices were associated with BOLD state descriptor and functional connectivity strength in each group.

**Results:**

The migraine without aura group showed significantly better performance on statistical learning compared to healthy controls. Based on the clusterability, low and high variability BOLD states were identified. Lower uptime in high variability states was found in migraine patients compared to healthy controls (*p* < .05). Differing correlation strength between the learning indices and the temporal state descriptors was found across groups. In addition, varying functional network connectivity strength in the low variability state correlated with the learning indices differently in migraine patients and healthy controls.

**Conclusion:**

Our results indicate that low variability BOLD states are associated with enhanced statistical learning in migraineurs. Furthermore, subtype-specific improvements in implicit pattern learning were observed, which may reflect adaptive network dynamics. These findings suggest that BOLD variability is a reliable functional imaging biomarker to better understand memory-related alterations in migraine.

## Introduction

Migraine is a primary headache disorder affecting approximately 10% of the population. Its hallmark symptoms include episodic severe headaches accompanied by nausea, photophobia, or vomiting. Patients are typically classified based on the presence or absence of transient neurological disturbances known as aura. Although numerous functional neuroimaging studies shed light on widespread alterations in brain activity and functional connectivity among individuals with migraine, the exact functional changes might remain incompletely understood [1, 2]. In addition to the clinical symptoms of migraine patients, the impact of the disease on cognition and learning ability has also been studied [3].

Alterations in functional connectivity patterns during the ictal and interictal phases were shown in migraine patients. Following the cyclic changes over time, the strength of the connectivity shows decreasing tendencies in the ictal phase, while during prodromal and interictal phases, strengthening functional connectivity can be observed [4]. In the pre-ictal and peri-ictal phases, abnormally increased functional connectivity strength forms in almost all brain networks, bilaterally [5]. Several processes may underlie the changes in network-level functional connectivity patterns that include disrupted excitation-inhibition coupling [6], cortical spreading depression [7], or altered levels of different neurotransmitters and neuromodulators [8]. Consequently, using resting-state functional imaging (rs-fMRI), the abnormalities in the amplitude of low-frequency fluctuations and in the functional connectivity patterns can be measured [9, 10]. Recent studies suggested that in rs-fMRI, the moment-to-moment blood-oxygen-level-dependent (BOLD) signal variability is associated with the severity of clinical symptoms and pain sensitivity [11, 12]. As a variance-based measure, using temporal BOLD signal variability seemed to be a robust and reliable measure in understanding individual differences in fMRI research [13].

Parallel to the clinical symptoms, several cognitive domains, including attention, verbal and visuospatial memory, information processing speed, and executive functions, are affected in migraineurs as well [14]. Associated with the cognitive impairment, alterations in network-level, as well as in intra-regional functional connectivity, were already identified [15, 16]. Although our knowledge about the cognitive impairment and general neuropsychological state of migraineurs is relatively well established, the impact of migraine on their implicit statistical learning skill is less well understood. Implicit learning is a fundamental process in cognition, and as opposed to explicit learning, it does not involve awareness, and it is not hypothesis-driven [17]. The extent of implicit statistical learning can be well quantified using the serial reaction time task, where participants’ decreasing reaction time implicates the implicit learning rate of a hidden sequence [18]. Although a small portion of the article focuses specifically on the learning abilities of migraine patients, their results show more often better associative or visuospatial learning skills compared to healthy controls [19] et al., [20]).

In this work, we investigate whether resting-state BOLD signal variability in episodic migraine patients with and without aura during the interictal state is associated with their implicit statistical learning skills. In addition, we compare the rate of implicit statistical learning of migraineurs and its association with resting-state BOLD signal variability to healthy controls. Based on prior evidence, we hypothesized that statistical learning performance would differ between migraine and healthy participants, and that these differences would be explained by distinct patterns of BOLD signal variability across groups.

## Materials and methods

### Participants

In this study, 28 episodic migraine patients–14 with aura (MwA) and 14 without aura (MwoA)–and 22 healthy controls (HC) were enrolled. The sample size was determined by the number of eligible participants who volunteered to take part in this study. All migraine patients were recruited at the Headache Outpatient Clinic of the Department of Neurology, University of Szeged, Hungary. Patients were diagnosed with episodic migraine according to the International Headache Society criteria. Exclusion criteria were the presence of any other neurological or psychiatric condition. 14 out of 28 migraineurs experienced aura symptoms. Patients were headache-free at least 48 h prior to the scanning and cognitive testing sessions. All participants provided their written informed consent according to the Declaration of Helsinki. The study was approved by the local ethics committee of the University of Szeged, Hungary (ref. ID: 166/2020-SZTE RKEB).

### Scanning protocols

Migraine patients and healthy controls were scanned on a 3T GE MR750W Discovery MRI scanner. Structural T1-weighted scans (3D T1-weighted MPRAGE sequence, TR: 7.6 ms, TE: 3 ms, matrix: 240 × 240, slice thickness: 1 mm, flip angle: 8°, whole brain coverage, isovoxel) and resting-state functional MRI scans (T2*-weighted GE-EPI sequence, TR: 2500 ms, TE: 27 ms, in-plane resolution: 3 mm × 3 mm, FOV: 288 mm x 288 mm, matrix 96 × 96, slice thickness: 3 mm, flip angle: 81°, 44 axial slices providing whole-brain coverage, interleaved acquisition scheme, 168 volumes, 7-minute long scans) were acquired. Patients were asked to lie awake in the scanner with their eyes open.

### Alternating serial reaction time task

**Fig. 1:**
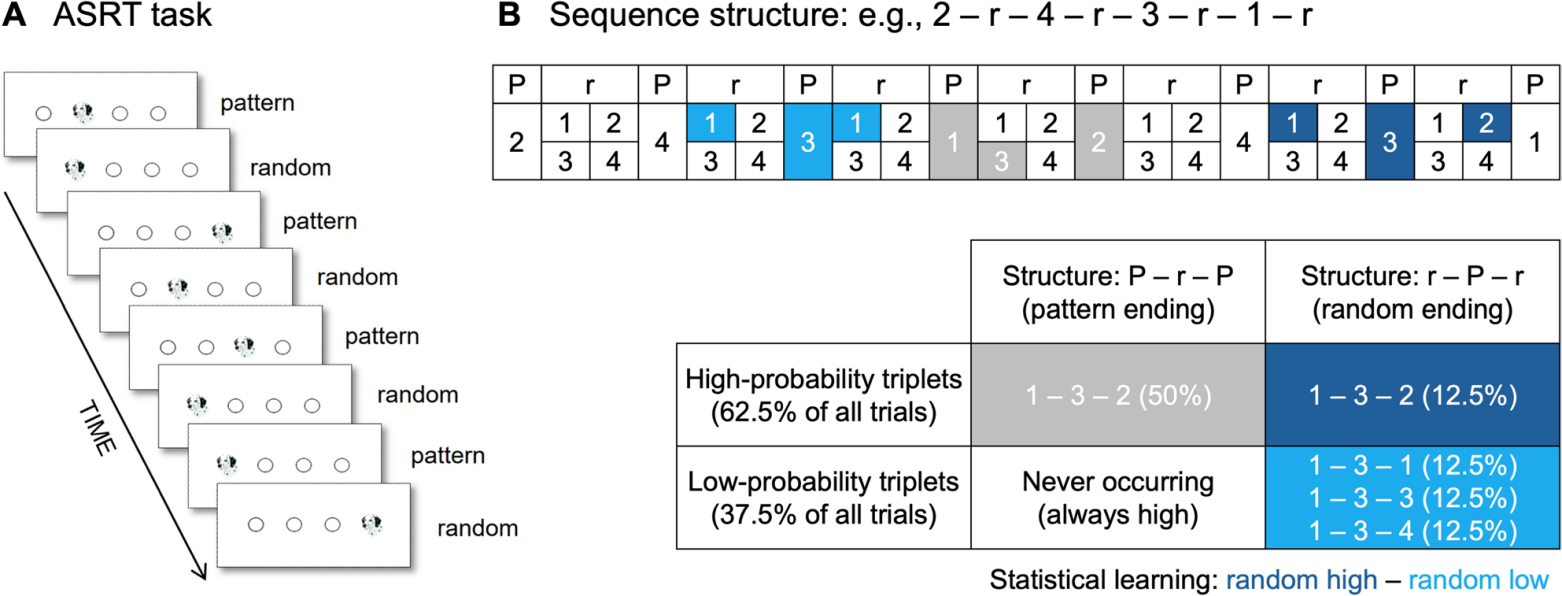
The Alternating Serial Reaction Time (ASRT) task. (**A**) Pattern and random trials alternated, using identical dog head images across all trials. Stimulus location defined trial type: pattern trials followed a predetermined sequence, while random trials used unpredictable locations among four options. (**B**) An example of the sequence structure, where numbers mark fixed stimulus locations of pattern trials, and ‘r’ indicates random locations. Due to the alternating design, certain trials can be identified as the third element of a triplet using a moving window. High-probability triplets include both pattern-ending (grey, 50%) and random-ending (dark blue, 12.5%) types, together making up 62.5% of all trials. Low-probability triplets are all random-ending (light blue) and account for the remaining 37.5%. Statistical learning is measured by comparing performance on random high (dark blue) and random low (light blue) trials

To assess implicit statistical learning, the Alternating Serial Reaction Time (ASRT) task was used [21]. Participants viewed a visual stimulus (a drawing of a Dalmatian dog’s head) on a computer screen (see Fig. 1A). The stimulus appeared within one of four horizontally arranged circles, each corresponding to a specific response key on a QWERTZ keyboard (Z, C, B, M, from left to right). Participants were instructed to press the key corresponding to the location of the stimulus using their left and right index and middle fingers while trying to be as quick and accurate as possible. The stimulus remained on the screen until a response was made, after which a 120 ms response-to-stimulus interval preceded the subsequent trial. Participants were informed that the task was a simple reaction-time task and were unaware of the underlying sequential structure. The stimuli stream followed an 8-element probabilistic sequence, in which every second trial was part of a predetermined sequence, while the intervening trials were randomly selected (e.g., 2–r–4–r–3–r–1–r) (see Fig. 1B). The probabilistic sequence repeated 10 times within a block, resulting in 80 trials per block, and the task consisted of 25 blocks in total. The probabilistic sequence structure allowed for the classification of trials based on two distinct aspects. Sequence-based categorization distinguished whether a trial was part of the predetermined sequence (pattern trial) or a randomly assigned position (random trial). On the other hand, probability-based categorization determined whether a trial belonged to a high-probability triplet or a low-probability triplet. A triplet refers to a chunk of three consecutive trials, where in high-probability triplets, the third element could be predicted from the first one with a probability of 62.5%. In contrast, in low-probability triplets, the third element was less predictable, occurring with only 37.5% probability. Given this structure, trials could be classified into three types: (1) pattern elements (which were always high-probability), (2) random high-probability trials, and (3) random low-probability trials (see Fig. 1B). To ensure that sequence-based effects did not confound statistical learning measurements, only random trials were included when comparing performance for high- and low-probability trials. This approach allowed us to assess pure statistical learning, as the two trial types (random high and random low) shared the same sequence properties but differed in their statistical properties. Higher accuracy and faster reaction times (RTs) for random high-probability triplets compared to random low-probability triplets reflect participants’ increasing sensitivity to the underlying statistical structure.

### Image preprocessing

Resting-state functional images were preprocessed with FSL FEAT v.6.0.0 implemented in the FSL software package (v.6.0.7.9) [22]. The first 4 volumes were removed to allow for steady-state magnetization. As implemented in FSL MCFLIRT, motion correction was performed with a rigid-body realignment procedure. Non-brain tissues were removed from the images using FLS BET [23]. Images were slice time corrected and then spatial smoothing was applied with a Gaussian kernel of 6 mm full-width-at-half-maximum. Functional images were aligned to standard MNI template space through a two-stage registration process. First, functional images were registered boundary-based to structural scans that was followed by non-linear spatial normalization to the standard 2 mm MNI space using FSL FNIRT. For better correction of the motion-related artifacts, ICA-AROMA was applied [24]. After motion correction, nuisance regression was applied to remove the signal of the cerebrospinal fluid and white matter, respectively. Finally, a high-pass filter was applied to resulting timeseries with a 0.008 Hz cutoff.

### Calculation of BOLD signal variability

After functional images were preprocessed, the 200-parcel resolution Schaefer-atlas was used for parcellation [25]. In each parcel, the mean timeseries of voxels were extracted, then demeaned and normalized to unit standard deviation. This step ensured that differences in BOLD signal variability was not influenced by the BOLD signal amplitude differences as it was reported in previous migraine studies [26]. BOLD signal variability was calculated as the successive squared difference (SSD) of signal intensity at each timepoint [13]. According to our previous study, we omitted the temporal averaging step in the denominator for making temporal clustering feasible (described in the next section), resulting in time series equivalent to the squared first-order temporal derivative of the BOLD signal [27]. To facilitate readability, we will refer to BOLD signal SSD as BOLD signal variability (BOLD_SV_) in the manuscript.

### Calculating temporal states from BOLD_SV_

BOLD_SV_ timeseries were arranged in a timepoint by region matrix for each subject. To determine different BOLD_SV_ patterns, k-means clustering algorithm was applied on the temporally concatenated (8150 time points × 200 ROIs; 50 subjects × 163 TRs each) BOLD signal variability matrices. Classical K-means clustering was applied to the time points using Euclidean distance. We evaluated cluster solutions with K = 2–10. To select the optimal number of clusters, we computed several well-established validation criteria: the Calinski–Harabasz (CH) index, the average silhouette coefficient, the elbow method based on within-cluster sum of squares (WCSS), and the Gap statistic. For each K, k-means was repeated 50 times with random initializations, and the solution with the highest CH index was retained. The mean silhouette coefficient and WCSS were then computed from this best solution. Following clustering steps, in each derived temporal state, maximum, mean and median dwell time (DT) and fractional occupancy (FO) was calculated, as state descriptors. Dwell time indicates the time the given participant spent in a certain temporal state, while fractional occupancy is a ratio of the dwell time and the full duration of the scan [27].

**Fig. 2:**
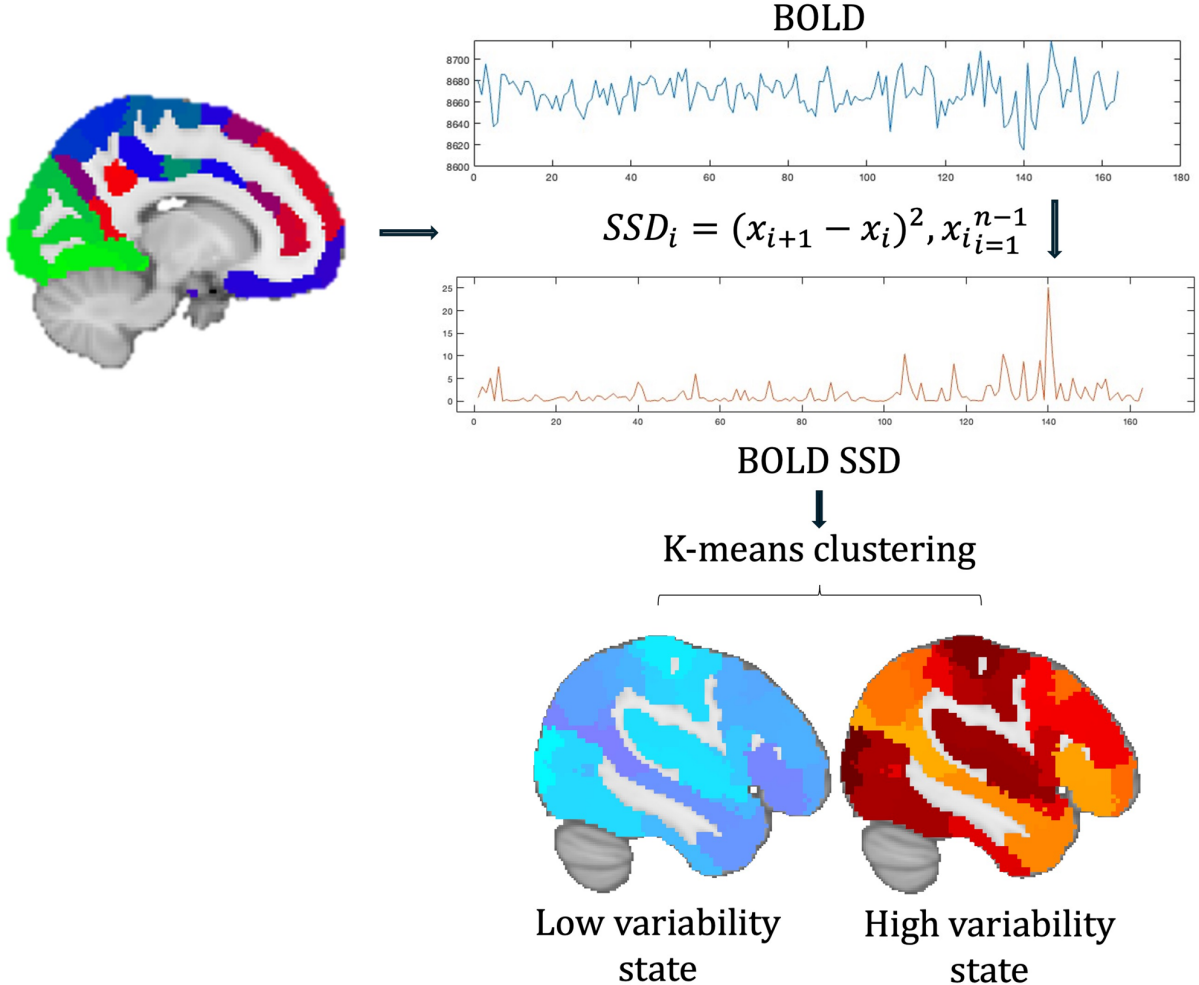
Schematic depiction of the analysis workflow. Using the 200 parcel Schaefer-atlas, frame wise BOLD SSD was calculated in each region as per the equation in the figure (where *i* denotes the time point, *x* denotes the normalized BOLD activity at the given time point and *n* denotes the total number of scans). Using K-means clustering on SSD time series temporal BOLD SSD states were derived

In addition, functional connectivity (FC) patterns derived from the different BOLD_SV_ states were compared. Region of interests (ROI) averaged BOLD_SV_ timeseries from the 200-parcel Schaefer-atlas were extracted, and inter-regional FC was calculated using Pearson’s correlation between regions during different BOLD_SV_ states after each region was assigned to 7 canonical resting-state network membership as it is indicated in the Schaefer-atlas, established on the Yeo-parcellation [25, 28]. Using non-parametric permutation test as implemented in FSL randomize, we analyzed whether the FC between groups and between states differs [29]. The design matrix also contained the age and gender as regressors. The images were corrected for multiple comparison using Family-wise error rate correction (FWE) and thresholded at *p* < .05. Schematic depiction of the analysis workflow is shown on Fig. 2.

### Statistical analysis ASRT

Trials that exhibited pre-existing response tendencies (e.g., trills such as 1-2-1 and repetitions such as 2-2-2) were excluded from the analysis. Additionally, RTs were filtered using a fixed percentile-based threshold: values falling below the 2nd or above the 98th percentile of the RT distribution were removed. This procedure resulted in the exclusion of 3.94% (SD = 3.85%), 3.98% (SD = 1.63%), and 3.98% (SD = 2.59%) of trials in the HC, MwoA, and MwA groups, respectively. A one-way ANOVA on exclusion rates revealed no significant group differences (F(2, 47) = 0.001, *p* = .99) while the Levene test indicated homogeneity of variances (*p* = .279).

The 25 blocks were divided into three larger bins: an early phase (Block 1–10) and a late phase of the task (Block 16–25) while keeping the middle phase (Block 11–15) separate. For each of these bins, the mean accuracy and RT were calculated separately for random high- and random low-probability trials. Accuracy-based statistical learning was quantified by subtracting the mean accuracy of random low-probability trials from that of random high-probability trials within each bin. For RT-based statistical learning, the mean RT of random high-probability trials was subtracted from that of random low-probability trials within the given bin. This resulted in three accuracy-based and three RT-based statistical learning indices for each participant, corresponding to the three bins of blocks. Overall accuracy-based and RT-based statistical learning scores, as well as difference scores (subtracting the learning index of the early phase from that of the late phase) were also calculated for each participant.

To compare statistical learning performance across the three groups, we conducted mixed-design ANOVAs. The dependent variables were either the accuracy-based or RT-based learning indices, while Group (MwoA vs. MwA vs. HC) served as a between-subjects factor, and Block Bin (Block 1–10 vs. Block 11–15 vs. Block 16–25) was included as a within-subjects factor. When conducting multiple comparisons, Bonferroni correction was applied when necessary. The significant threshold was set at *p* < .05. Effect sizes for the ANOVAs were reported using partial eta squared (η²p). We note that this approach does not fully capture the hierarchical structure of the ASRT data, which is a limitation of the current analyses.

Finally, non-parametric permutation test was applied to determine whether learning indices correlation with FC pattern differs between groups and temporal states ([MwoA] vs. [MwA] vs. [HC]). The design matrices contained the age, gender and the learning indices for each participant separately. The images were corrected for multiple comparison using FWE correction and thresholded at *p* < .05.

### Statistical analysis of state descriptors

State descriptors were tested for normality using Kolmogorov-Smirnov test and compared between groups (MwoA vs. MwA vs. HC) with ANOVA. Correction for multiple comparisons was performed using false discovery rate (FDR) correction using the Benjamini-Hochberg method. We used q = 0.05 as the FDR threshold. All p-values reported in the manuscript reflect FDR-corrected p-values. To determine pairwise group differences Tukey’s HSD post-hoc test was used. To calculate whether the correlation between state descriptors and accuracy-, and RT-based learning indices differs between groups (MwoA vs. HC; MwA vs. HC; MwoA vs. MwA), partial correlations were computed between the state descriptors and ASRT performance variables. Partial correlations were controlled for age, age², and gender. All correlations were calculated using Pearson’s *r* with listwise deletion. To quantify the uncertainty of each correlation estimate, we computed 95% confidence intervals analytically using the Fisher *z* transformation. To test whether the strength of correlations differed between the three groups, we used Fisher’s *r*-to-*z* transformation for independent samples. To control for multiple comparisons, all *p*-values from Fisher tests were corrected using the Benjamini–Hochberg False Discovery Rate (FDR) procedure. Two-tailed *p*-values were calculated from the standard normal distribution. We tested all 7 × 10 pairwise correlations for all three group contrasts (HC vs. MwA, HC vs. MwoA, MwA vs. MwoA), yielding 210 comparisons. Group-wise significant correlations were visualized with heatmaps, while between-group correlation differences were displayed using Fisher-*z* heatmaps (masked at FDR-corrected *p* < .05). For each significant between-group difference, scatterplots were generated using partial residuals after regressing out covariates.

## Results

All the demographic and main clinical data of all participants can be found in Table 1.

**Table 1:** Main demographic and clinical data of migraine patients and healthy controls

	Migraine without aura	Migraine with aura	Healthy controls	Comparison
Age (Median ± IR)	33.00 ± 17.75	31.00 ± 11.50	32.00 ± 18.75	*p* = .519
Gender (FM/M)	12/2	8/6	14/8	*p* = .227
Education (years, median ± IR)	16.50 ± 4.75	18.00 ± 5.50	17.00 ± 6.50	*p* = .506
HAM-D score (median ± IR)	5.00 ± 4.75	2.50 ± 3.25	3.00 ± 5.00	*p* = .382
Disease duration (years, median ± IR)	10.50 ± 18.25	16.00 ± 14.00	-	*p* = .466
Attack frequency (attacks/year, median ± IR)	30.00 ± 39.00	36.00 ± 30.00	-	*p* = .382
Allodynia scores (median, range)	0.5 (0–9)	0 (0–9)	-	*p* = .880
MIDAS score (median ± IR)	31.00 ± 30.50	29.50 ± 26.25	-	*p* = .289
Interval therapy	1 iprazochrome	3 beta-blocker	-	*p* = .149

### Enhanced accuracy-based statistical learning in migraine without aura

**Fig. 3:**
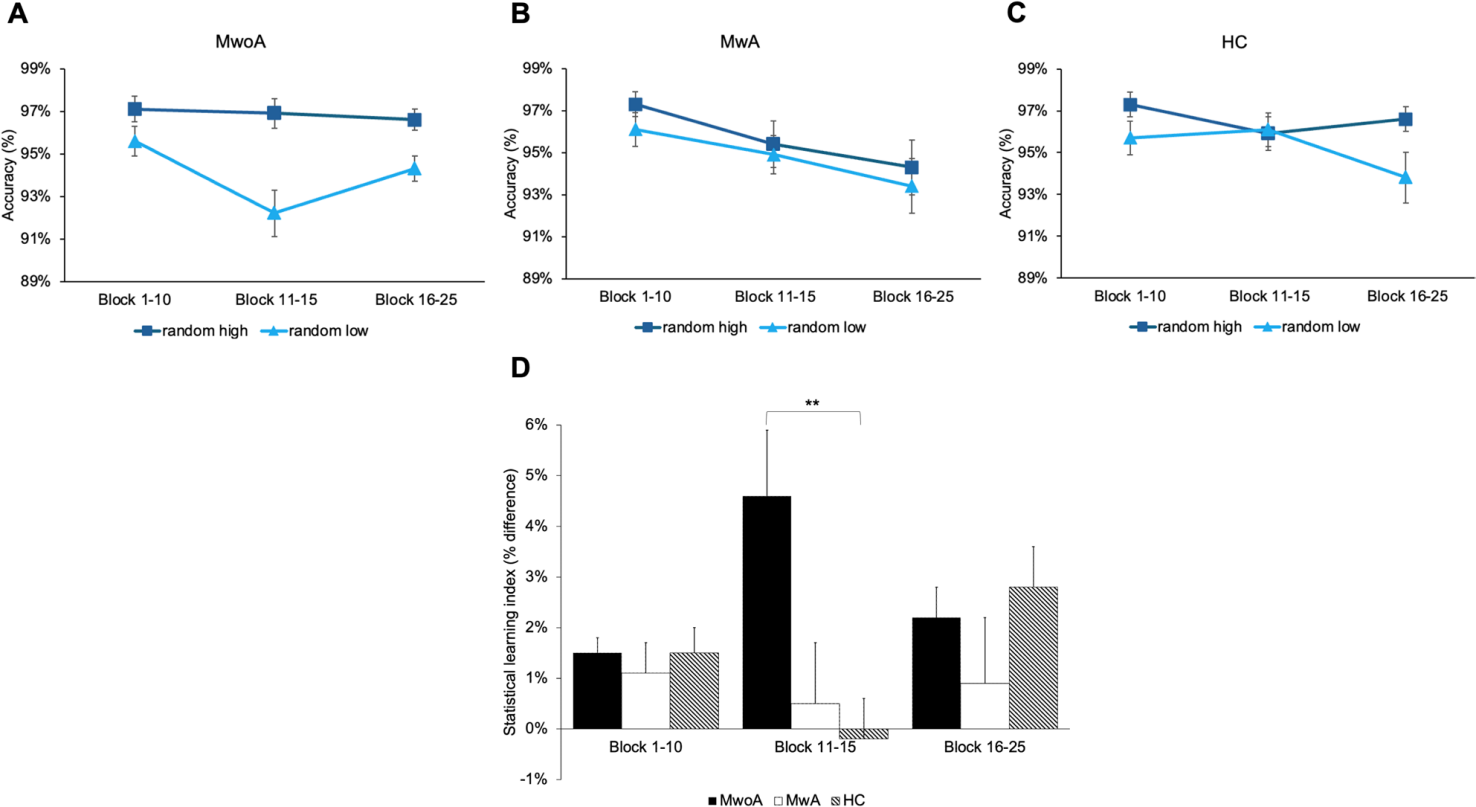
Accuracy-based statistical learning in the three groups. The y-axis shows accuracy in percentage, while the x-axis represents time across the three main phases of the task (early: Block 1–10, middle: Block 11–15, late: Block 16–25). Error bars represent SEM. (**A**) Accuracy comparison between random high and random low trials in the migraine without aura (MwoA) group. This group consistently maintains high accuracy on random high trials, while accuracy on random low trials dips during the middle phase. (**B**) Accuracy comparison in the migraine with aura (MwA) group. Accuracy on random high and low trials remains closely aligned throughout the task, with minimal difference. (**C**) Accuracy comparison in the healthy control (HC) group. The learning effect disappears in the middle phase as performance on the two trial types converges but re-emerges in the late phase. (**D**) Group comparison of accuracy-based learning across task phases, where a higher learning index indicates better learning. In the middle phase, the MwoA group performed significantly better than the HC group (*p* < .01)

A mixed-design ANOVA was conducted on the accuracy-based statistical learning index, with Group (MwoA vs. MwA vs. HC) as a between-subjects factor and Block Bin (Block 1–10 vs. Block 11–15 vs. Block 16–25) as a within-subjects factor. Mauchly’s test indicated a violation of the sphericity assumption (W = 0.542, *p* < .001); therefore, Greenhouse-Geisser correction was applied. The main effect of Block Bin was not significant, indicating that accuracy-based statistical learning did not change significantly over the course of the task across all participants (F(1.372, 64.487) = 0.388, *p* = .601, η²*p* = .008). However, the Group × Block Bin interaction revealed significant differences in the learning dynamics between groups F(2.744, 64.487) = 3.803, *p* = .017, η²*p* = .139) (see Fig. 3A-C). Post-hoc comparisons showed that this difference was driven by a significantly better performance of the MwoA group compared to HC during the middle phase of the task (i.e., Block 11–15) (t = 4.055, *p* = .003) (see Fig. 3D). The main effect of Group suggested a trend-level difference between groups, but this did not reach statistical significance (F(2, 47) = 2.821, *p* = .070, η²*p* = .107). Post-hoc comparisons further indicated that this trend was primarily due to the superior statistical learning performance of the MwoA group compared to the MwA group (t = 2.280, *p* = .082).

### Enhanced RT-based statistical learning in migraine without aura

**Fig. 4:**
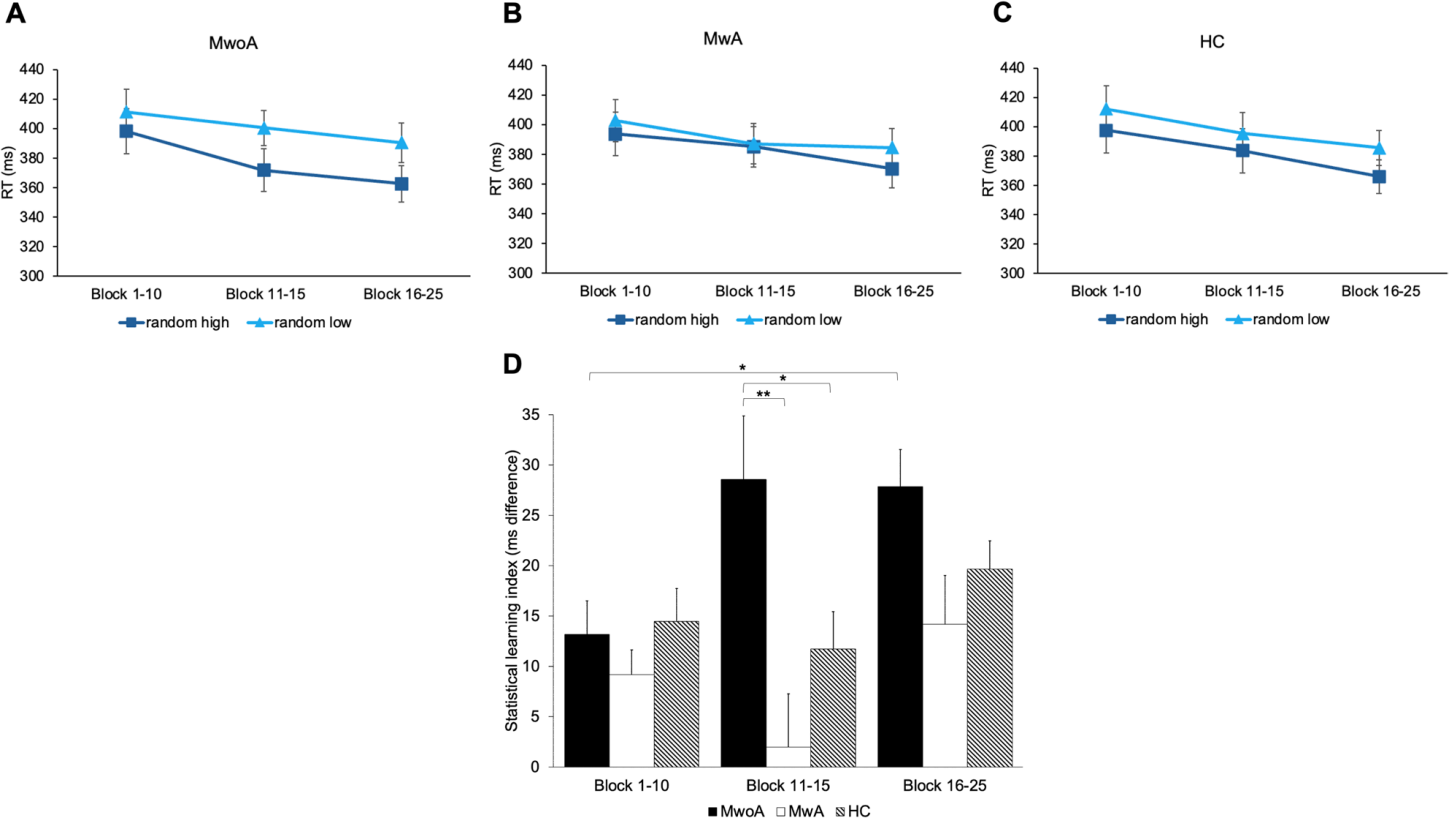
Reaction time-based statistical learning in the three groups. The y-axis displays reaction time (RT) in milliseconds, while the x-axis represents the progression of the task divided into three phases (early: Block 1–10, middle: Block 11–15, late: Block 16–25). Error bars represent SEM. (**A**) RT comparison between random high and random low trials in the migraine without aura (MwoA) group. This group showed the most pronounced learning effect, with the largest difference emerging in the middle phase of the task and persisting into the late phase. (**B**) RT comparison in the migraine with aura (MwA) group. The two trial types closely track each other throughout the task; learning diminishes markedly in the middle phase and only begins to re-emerge in the late phase. (**C**) RT comparison in the healthy control (HC) group. The difference between random high and low trials remains relatively stable across the task, with a slight improvement toward the end. (**D**) Comparison of RT-based learning across the three groups and task phases, where a higher learning index indicates better learning. The MwoA group outperformed both the MwA (*p* < .01) and HC (*p* < .05) groups in the middle phase of the task. Additionally, a significant difference between the early and late phases was observed only in the MwoA group (*p* < .05)

A mixed-design ANOVA was conducted on the RT-based statistical learning index, using Group (MwoA vs. MwA vs. HC) as a between-subjects factor and Block Bin (Block 1–10 vs. Block 11–15 vs. Block 16–25) as a within-subjects factor. Mauchly’s test of sphericity indicated that the assumption of sphericity was not violated for the within-subject factor (W = 0.898, *p* = .08); therefore, no Greenhouse-Geisser correction was applied. A significant main effect of Block Bin indicated that RT-based statistical learning improved over time as the task progressed (F(2, 94) = 4.557, *p* < .05, η²*p* = .088). The main effect of Group revealed significant differences in statistical learning performance between the groups (F(2, 47) = 5.810, *p* < .01, η²*p* = .198). Post-hoc comparisons showed that participants in the MwoA group exhibited significantly higher overall learning scores than those in the MwA groups (t = 3.405, *p* < .01) (see Fig. 4A-C). A significant Block Bin × Group interaction indicated that the time course of learning also differed between the groups (F(4, 94) = 2.633, *p* < .05, η²*p* = .101). Post-hoc comparisons revealed that the MwoA group performed significantly better than the MwA group during the middle phase of the task (Block 6–10; t = 3.519, *p* < .01) and also outperformed the HC group in the same period (t = 2.466, *p* < .05). Furthermore, the MwoA group was the only one to show a significant improvement in statistical learning from the early to the late phase of the task (t = -3.307, *p* < .05) (see Fig. 4D).

### Distinct low and high variability States alternate during rest

We evaluated K = 2–10 using multiple cluster-validation criteria. The Calinski–Harabasz index showed a clear maximum at K = 2 (CH = 408.6), followed by a monotonic decrease for higher K values (e.g., CH = 276.2 for K = 3 and CH = 93.1 for K = 10). The average silhouette coefficient was also highest at K = 2 (0.087) and declined steadily thereafter (e.g., 0.048 at K = 3, 0.018 at K = 10). The elbow plot of WCSS displayed a pronounced bend between K = 2 and K = 3, after which the decrease in WCSS became gradual and linear, consistent with a parsimonious 2-state solution. The Gap statistic showed only minimal variation across the tested range (1.85–1.86 for K = 2–10) with a shallow increase toward higher K and did not provide a clear low-K maximum. Given the strong and convergent evidence from the CH index, silhouette coefficient, and WCSS elbow, and the interpretability and stability of a 2-state model, we retained K = 2 as the optimal number of clusters for subsequent analyses.

**Fig. 5:**
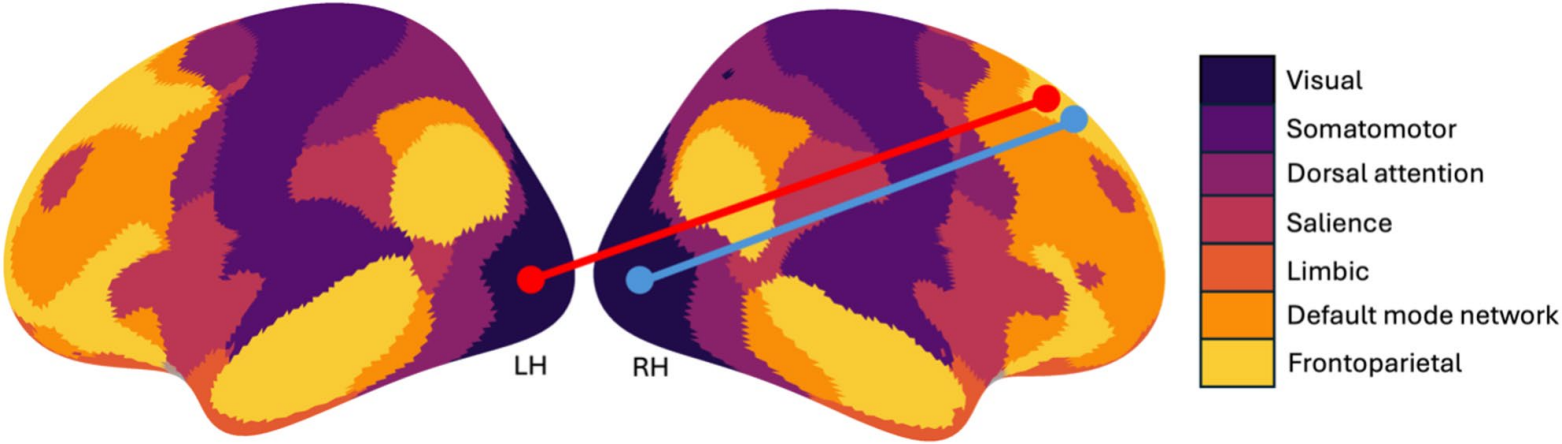
Group wise differences in functional connectivity. In low BOLD_SV_ state greater connectivity was observed between the right visual and right frontoparietal control networks (blue line) in HC compared to both the MwA and MwoA group as well. Likewise, in high BOLD_SV_ state greater connectivity was observed between the left visual and right frontoparietal control networks (red line) in HC compared to MwoA group. (LH: left hemisphere, RH: right hemisphere, based on FWE corrected *p*<.05 values)

The non-parametric permutation test revealed that the low variability state was associated with significantly stronger between-network connectivity in HC between regions of the right visual and right frontoparietal control networks compared to MwA and MwoA, respectively (see Fig. 5. blue line). Similarly, significantly stronger between network connectivity in high variability state was observed between the left visual and right frontoparietal control network in HC compared to MwoA as well (see Fig. 5. red line).

### Migraineurs spend less time in the high variability state

**Fig. 6:**
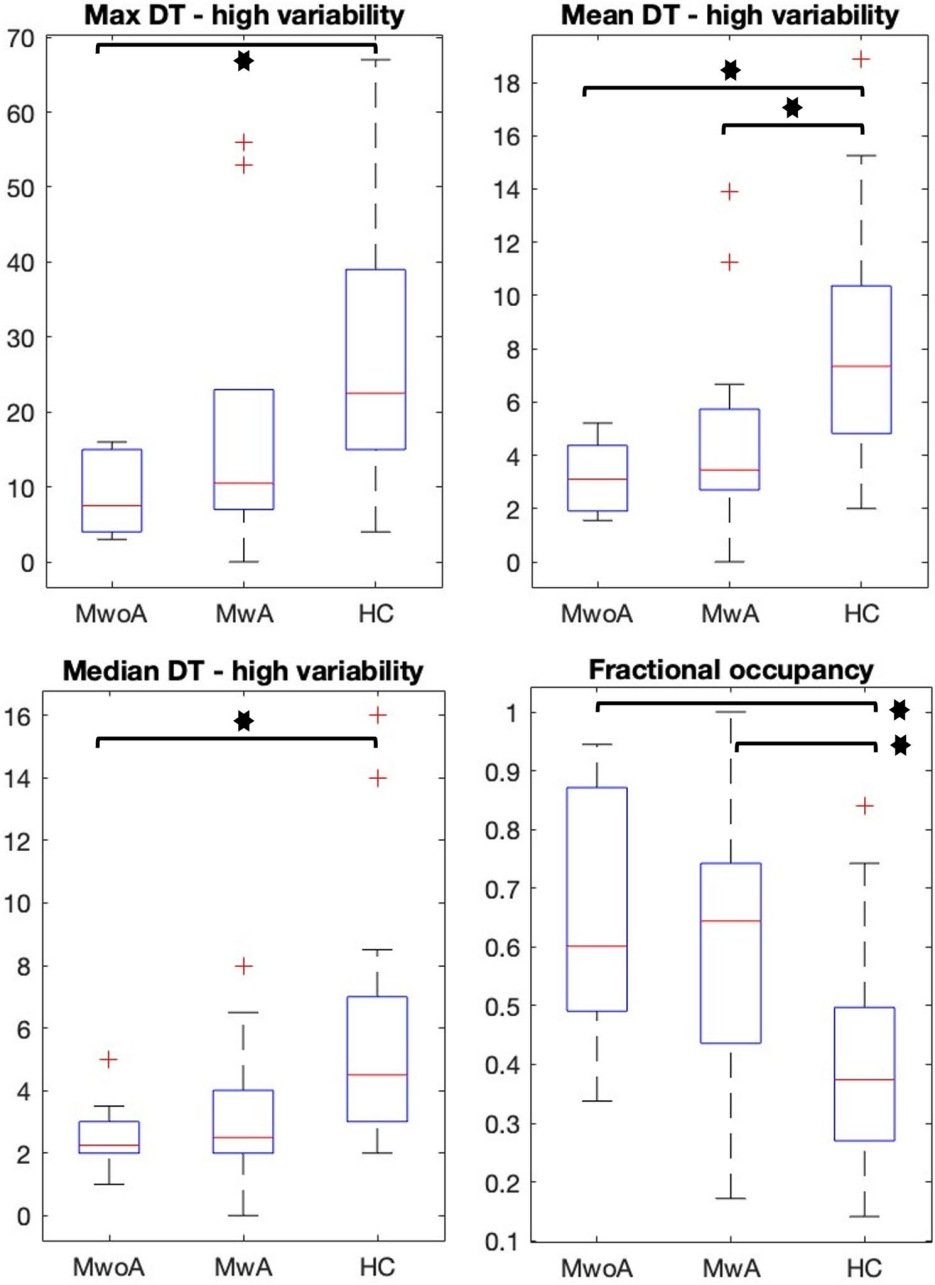
Group comparison of state descriptors. The y-axis shows either the ratio of fractional occupancy or the dwell time in seconds, while the x-axis represents groups (MwoA: migraine without aura; MwA: migraine with aura; HC: healthy controls). The boxplots show that migraineurs spend significantly less time in the high variability state compared to healthy controls but spend more time fractionally in the low variability state. (FO: fractional occupancy; DT: dwell time; MwoA: migraine without aura; MwA: migraine with aura; HC: healthy controls, * indicates FDR corrected *p* < .05 values)

No difference between groups (MwoA vs. MwA vs. HC) was shown in the maximum dwell time (F(2,47) = 2.38, *p* = .144), in the mean dwell time (F(2,47) = 1.61, *p* = .243) and in the median dwell time (F(2,47) = 1.41, *p* = .252) spent in the low variability state. Contrary, ANOVA showed significant group differences in the maximum dwell time (F(2,47) = 6.903, *p* < .01) spent in high variability state. The Tukey’s HSD post-hoc test showed that only the MwoA group differed significantly from the HC group (*p* < .003). Also, migraineurs had less mean dwell time (F(2,47) = 7.694, *p* < .009) compared to HC. The post-hoc test revealed, that both the MwoA group (*p* < .001) and MwA group (*p* < .041) differed from HC, but no difference between MwoA and MwA groups was shown. Furthermore, migraineurs groups significantly differed in the median dwell time (F(2,47) = 5.449, *p* < .01) spent in the high variability state compared to HC. The post-hoc test showed only significant group differences between MwoA and HC groups (*p* < .008). Finally, significant group difference was revealed in the fractional occupancy (F(2,47) = 6.780, *p* < .009) ratio, where both the MwoA group (*p* < .004) and MwA group (*p* < .026) differed from HC. Important to note, that fractional occupancy was calculated only in the low variability state, due its inverse is the fractional occupancy ratio in the high variability state. Thus, no differing but inverse statistical result could be seen. Between-group differences of the state descriptors are depicted on Fig. 6., and all p-values indicated in this interpretation were FDR corrected.

### Group differences in learning indices and state descriptor correlations

**Fig. 7:**
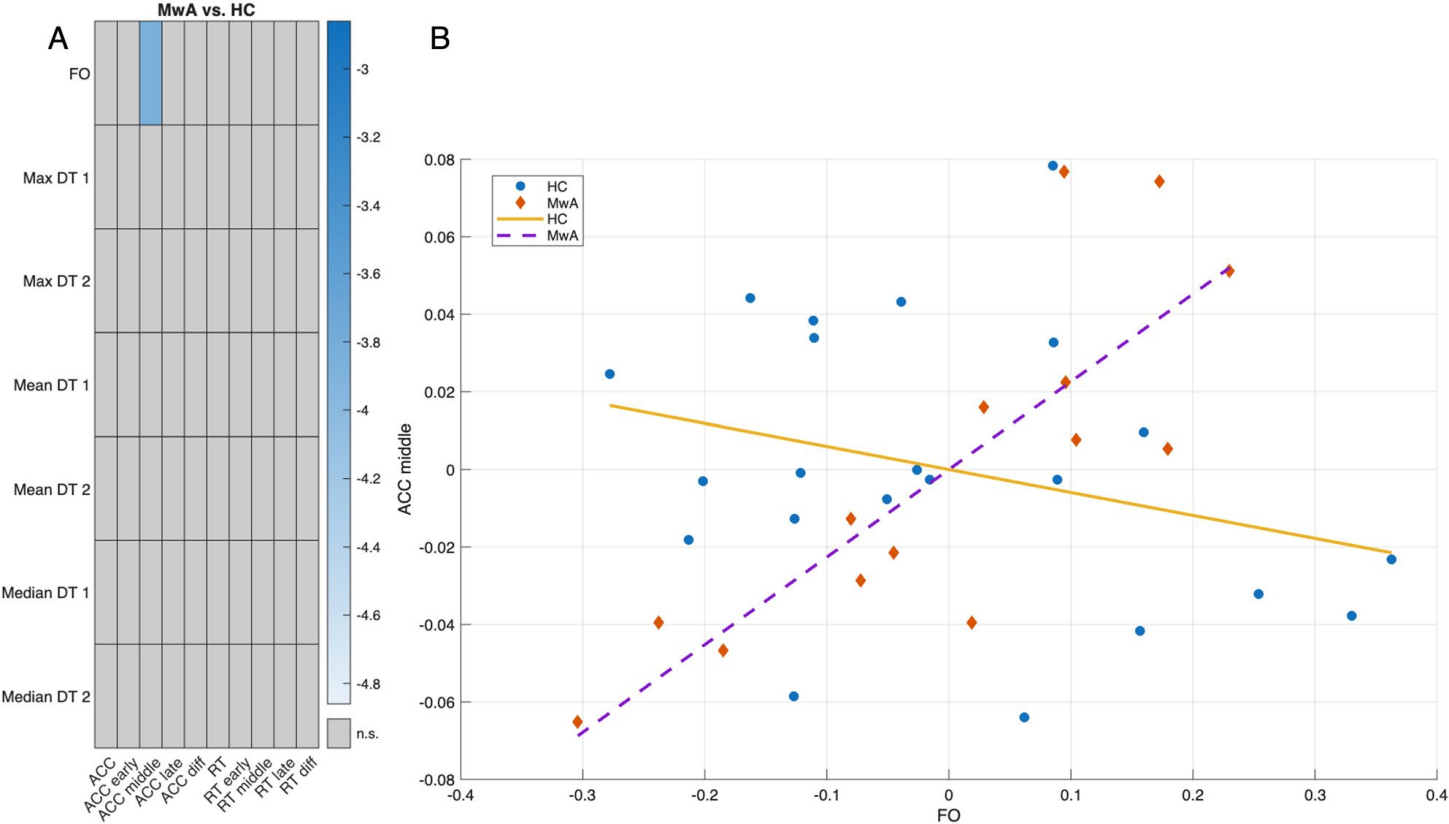
Groupwise differences in learning indices and state descriptor correlations. **A**) depicts the only significant MwA and HC group difference between FO and accuracy in early block bin (black rectangle) after correction for multiple comparison. In the heatmap, light-blue rectangle denotes FWE corrected *p*<.05 values **B**) depicts the extent and direction of correlation differences between FO and accuracy in early block bin between MwA (blue datapoints - blue regression line) and HC (red datapoints - red regression line) group (FO: fractional occupancy; ACC: accuracy; RT: reaction time; DT: dwell time; MwA: migraine with aura; HC: healthy controls)

Fisher’s *r*-to-*z* comparison revealed that several state–behavior associations differed significantly between groups after FDR correction. One robust effect was observed for the relationship between FO and accuracy measured in the middle block bin. Healthy controls showed a small, non-significant negative partial correlation (*r* = − .289, 95% CI = − 0.634 to 0.151), whereas MwA group exhibited a strong positive association (*r* = .823, 95% CI = 0.518 to 0.942). The difference between these correlations was statistically significant (Fisher *z* = − 3.86, *p* = 1.1 × 10⁻⁴, *p* (FDR)= 0.024). This indicates that the coupling between FO dynamics and middle phase accuracy task performance is substantially stronger in MwA than in healthy controls (see Fig. 7A and B.).

### Learning phase dependent pattern changes in FC between migraineurs and controls

Using non-parametric permutation testing with appropriate FEW corrections for multiple comparisons, no interaction between functional connectivity differences in HC vs. MwoA, HC vs. MwA and MwoA vs. MwA and learning-related indices, such as accuracy rate, reaction time, and block-wise learning phases (early, middle, late) was found.

## Discussion

In this study, we investigated the relationship between resting-state BOLD temporal alterations and implicit learning skills in migraine patients and healthy controls. We found that MwoA exhibited higher accuracy- and reaction time-based learning indices compared to MwA and controls. The temporal alterations of BOLD variability were classified into low and high variability states. Group comparisons revealed that both MwoA and MwA spent significantly less time in the high BOLD variability state, though not in the low BOLD variability state, relative to controls. A higher fractional occupancy rate of the low BOLD variability state was associated with increased accuracy in MwA compared to controls. Across both states, higher functional connectivity was found between the visual and frontoparietal control networks in HC.

Implicit statistical learning is a cognitive process whereby individuals unconsciously perceive and internalize patterns, structures, and regularities in sensory inputs without explicit awareness or instruction [30]. Surprisingly, in our study, MwoA patients demonstrated better statistical learning performance than HCs in terms of both accuracy and reaction time during the middle block bins of the task, respectively. Although many studies have explored implicit learning, only a few have investigated its modulation in migraine. For example, in associative equivalence learning, migraineurs showed decreased performance in the association phase, but no significant differences in the test phase, compared to controls. Generalization of learned associations to novel stimuli was impaired in migraineurs, indicating potential hippocampal dysfunction [31]. The association phase involves is primarily dependent on the basal ganglia-frontal cortex loop, whereas the test phase relies more heavily on hippocampal functioning. However, when the task was less semantically demanding, migraineurs did not differ from controls in the number of trials required to learn associations or in error rates. In another associative pair learning study, migraineurs even outperformed the control group, and showed enhanced performance in executive and motor skills as well [32, 33]. One possible explanation, as the authors suggested, is the development of cognitive adaptation to maintain performance, potentially mediated by functional alterations in the basal ganglia and frontal cortices loop, as well as the hippocampus [34]. Another potential mechanism behind the superior performance is improved cerebral vascularization and increased cerebral blood flow during the interictal phase, which may serve as a compensatory neurovascular mechanism to preserve cognitive function [33]. Moreover, enhanced multisensory processing in migraineurs may reflect sensory hypersensitivity, possibly due to cross-modal sensitization [32]. In contrast, prospective memory studies in MwoA patients have found impaired performance on time-based tasks, possibly due to disrupted coordination between intention formation and execution [35].

The contradictory results found in previous studies on how migraine impacts memory functions raise the question of whether specific underlying functional patterns could be identified. Although the general cognitive status of migraineurs, measured with the Montreal Cognitive Assessment, was found to be lower than that of controls and was predicted by the functional connectivity of the hippocampus-amygdala transition area, the effect of migraine on implicit statistical learning skills remains less well understood [36]. In a working memory (N-back) task, lower scores and higher activation were observed in the frontal pole and orbitofrontal cortex during the ictal phase—regions known to be involved in both inhibitory control and pain processing [37]. During probabilistic category learning, activation of the prefrontal, parietal, and occipitotemporal cortices was found [38]. However, only one task-based fMRI study has directly investigated the neural substrates of implicit statistical learning in healthy individuals. This study aimed to uncover activation patterns that contribute to the learning of predictable versus unpredictable statistical regularities. They found that not only specific brain activations but also the recruitment of prior knowledge contributed to learning, involving distinct neural pathways. In both conditions, activity of the bilateral insula and right inferior frontal gyrus was found. When comparing conditions, activation of the right globus pallidus and putamen was linked to predictable learning, while the activation of the right hippocampus was associated with unpredictable statistical learning. Taken together, the authors identified distinct brain activation patterns that support both the acquisition and maintenance phases of implicit statistical learning [39]. On the basis of the current concept detailed in the systematic review of David and colleagues, impaired or altered memory function in migraine may be explained by overlapping network hubs involved in both memory and pain processing. These shared hubs include both subcortical (e.g., hippocampus, amygdala, insula) and cortical areas (e.g., prefrontal, parietal, and temporal cortices). Supporting this notion, non-invasive magnetic stimulation applied to the dorsolateral prefrontal cortex exerted an analgesic effect via top-down inhibitory modulation on the midbrain-thalamic-cingulate loop [40]. In line with this, Liu and colleagues [41] demonstrated that focal hubs involved in pain processing may gradually evolve into a globally synchronized hub community over time. The resulting loss of normal topological network organization may impair connections between functionally diverse regions, thereby compromising memory functions through widespread dissemination of pain-related signals [41].

To further understand how migraine attacks and consequently the experienced pain influences functional brain connectivity between networks, a number of neuroimaging studies have been conducted. Regarding the central executive network, decreased functional connectivity (FC) has been reported in the fronto-parietal network hubs, including the middle frontal gyrus and dorsal anterior cingulate cortex in MwoA patients, despite no measurable differences in executive functions compared to controls [42]. A meta-analysis revealed reduced FC in prefrontal regions that serve as key hubs on both cognition and pain regulation within the default mode network (DMN). In addition, altered FC patterns were found occipital areas that are related to migraine due to their involvement in cortical spreading depression or hyperexcitability. Altered connectivity between the amygdala and middle occipital gyrus is argued to be associated with photophobia and headache severity in the interictal phase. Furthermore, reduced connectivity with the inferior parietal gyri may contribute to disruptions in memory formation and pain modulation [43]. Compared to HCs, migraineurs showed widespread bidirectional FC alterations in the fronto-parietal network bilaterally. Such disruptions may underlie attentional deficits previously reported in migraine, potentially contributing to impairments in higher-order cognitive processes, including working memory and decision making [44].

BOLD signal variability was previously thought to be sensitive not only to neural activity but also to physiological fluctuations such as respiration or cardiovascular rhythms. However, recent studies demonstrated that BOLD signal variability persists even after correcting for the aforementioned factors, and is reliably associated with attentional control, episodic memory, or executive functions [45, 46]. Although numerous studies investigated how BOLD signal variability is influenced by factors such as age, cognitive load, psychiatric diseases, or Alzheimer’s disease, less research addresses its role in migraine or its association with memory functions [47–51]. In our previous work, we found that migraineurs spend significantly more time in a low BOLD_SV_ state that was associated with greater disease severity [46]. Interestingly, regardless of episodic or chronic migraine status, greater BOLD variability was observed in the ascending trigeminal somatosensory pathway, whereas decreased BOLD variability was found in the top-down pain modulatory areas. The authors propose that higher variability may reflect better adaptive functioning through more balanced excitatory-inhibitory synaptic activity but may also indicate pathological amplification of sensory or pain-related signals. In addition, lower variability was associated with reduced thermal pain thresholds, possibly due to a deviation from the optimal noise level necessary for efficient neural processing [52]. In line with these findings, a systematic review by Schramm et al. [53] reported decreased BOLD variability of the insula, while increased BOLD variability of the trigeminal areas, hippocampus, and thalamus [53].

In our recent study, we found that greater time spent in the low BOLD_SV_ state was associated with increased disability [46]. However, in this cohort, MwoA and MwA spent significantly less time in high BOLD variability state, but not in low BOLD_SV_ state, compared to HCs. It is important to note that the two cohorts differed in key demographic and clinical characteristics, with higher attack frequency, age, and disease duration featured in this study. Previous studies have shown that BOLD_SV_ generally decreases with age across most resting-state networks, except for the salience network [48]. However, our understanding of how migraine alters the age-related trajectory of BOLD_SV_ remains limited [48]. Nevertheless, we found that a higher fractional occupancy rate of the low BOLD_SV_ state was associated with increased accuracy in MwA patients compared to controls. In other words, while healthy individuals seem to benefit from spending more time in the high BOLD_SV_ state, MwA patients achieve better performance when spending rather more time in the low BOLD_SV_ state. These findings align with the hypothesis that, although high BOLD variability typically supports adaptive cognitive functioning by balancing inhibitory and excitatory processes, in migraine it may simultaneously enhance sensitivity to pain-related sensory input. Thus, the low BOLD variability state may serve as a compensatory mechanism that helps mitigate this amplification. Notably, in both BOLD_SV_ states, HCs exhibited stronger FC between the visual and frontoparietal control networks.

At the between-network level, previous studies have reported widespread disruption of FC among migraineurs. In particular, disruption of FC between the visual cortex and other large-scale networks—such as the central executive, sensorimotor, or DMN—has been frequently observed, likely due to the high prevalence of visual symptoms in migraineurs [54]. Consistent with these findings, our results further support the concept that migraine affects the large-scale brain network functionality through shared network topologies.

## Conclusion

In this work, we demonstrated that migraine patients without aura performed better in an implicit statistical learning task compared to controls. Altered functional connectivity patterns in migraineurs were also revealed. Deriving resting state temporal BOLD signal variance states, we demonstrated that migraineurs spend less time in the high variability state.

### Limitations

Our study clearly has limitations. Firstly, the moderated sample size may have limited the statistical power and the generalizability of our findings. Although our primary behavioral analyses used aggregated data in mixed-design ANOVAs, this approach simplifies the hierarchical structure of the ASRT task by collapsing trial-level observations to the subject level. Such aggregation might reduce within-subject variability and assumes independence between subjects, which may limit sensitivity to subtle learning dynamics. A further limitation relates to the clustering approach. While our classical k-means clustering approach yielded a clear and interpretable two-state solution, alternative clustering frameworks may capture additional structure that might not be detectable with the present approach.

## Data Availability

The dataset analyzed in this study is available from the corresponding author on reasonable request after consideration by the local ethics committee.
